# The long non-coding RNA CRNDE acts as a ceRNA and promotes glioma malignancy by preventing miR-136-5p-mediated downregulation of Bcl-2 and Wnt2

**DOI:** 10.18632/oncotarget.21513

**Published:** 2017-10-04

**Authors:** Dong-Xue Li, Xiao-Rui Fei, Yong-Fei Dong, Chuan-Dong Cheng, Yang Yang, Xue-Fei Deng, Hai-Liang Huang, Wan-Xiang Niu, Chen-Xu Zhou, Cheng-Yu Xia, Chao-Shi Niu

**Affiliations:** ^1^ Shandong University, Jinan, Shandong, China; ^2^ Department of Neurosurgery, Anhui Provincial Hospital Affiliated to Anhui Medical University, Hefei, Anhui, China; ^3^ School of Basic Medical Sciences, Anhui Medical University, Hefei, Anhui, China

**Keywords:** CRNDE, glioma, miR-136-5p, Bcl-2, Wnt2

## Abstract

The colorectal neoplasia differentially expressed (CRNDE) gene encodes a long non-coding RNA (lncRNA) that is the most unregulated among 129 lncRNAs differentially expressed in gliomas. In this study, we confirmed high CRNDE expression in clinical glioma specimens and observed through experiments in human glioma cell lines a novel molecular mechanism by which CRNDE may contribute to glioma pathogenesis. By inducing or silencing CRNDE expression, we detected a positive correlation between CRNDE levels and the proliferative, migratory, and invasive capacities of glioma cells, which were concomitant with a decreased apoptosis rate. Our experiments also suggest that these effects are mediated by downregulation of miR-136-5p, which correlated with the glioma WHO grade. Based on predicted CRNDE/miR-136-5p/mRNA interactions, both the mRNA and protein expression analyses suggested that miR-136-5p-mediated repression of Bcl-2 and Wnt2 underlies the pro-tumoral actions of CRNDE. We therefore propose that CRNDE functions as a competing endogenous RNA (ceRNA) that binds to and negatively regulates miR-136-5p, thereby protecting Bcl-2 and Wnt2 from miR-136-5p-mediated inhibition in glioma.

## INTRODUCTION

Gliomas, the most common and aggressive type of primary malignant tumors in the adult human central nervous system, are characterized by difficulty in early diagnosis and extremely poor prognosis, especially at advanced stages and grades. Malignant gliomas infiltrate the brain parenchyma as they grow and commonly recur after surgery, radiotherapy, and/or chemotherapy. Since the prognosis of patients suffering from glioma remains poor despite newer and targeted therapies, it is crucial to understand the pathogenic mechanism of gliomas and to identify dysregulated genes to define novel biomarkers and relevant therapeutic targets.

Non-coding RNA (ncRNA) has attracted much attention due to increasing evidence indicating that its dysregulation is associated with a variety of cellular processes such as proliferation, migration, invasion, and apoptosis both in normal and neoplastic cells [1]. NcRNA subtypes include small RNA (sRNA; <200nt), comprising microRNA(miRNA) and small interfering RNA(siRNA), and long non-coding RNA (lncRNA; >200nt). In recent years, interest has been redirected from miRNA and siRNA to lncRNA, which is found in greater quantities and variety. LncRNAs have specific secondary structures that possess multiple binding sites for proteins, DNA, or RNA. In the last two cases, interaction occurs through the complementary base pairing principle, globally conforming a lncRNA-mediated gene expression regulatory network. In recent years, evidence has indicated that differentially expressed lncRNAs are closely associated with tumor initiation and development [2].

The colorectal neoplasia differentially expressed(CRNDE) gene, initially identified in colorectal cancer, is located on chromosome 16 and encodes a lncRNA that was proposed to serve as a serum-based diagnostic and prognostic tumor biomarker [3, 4]. CRNDE is also significantly upregulated and exerts oncogenic functions in diverse solid and hematological cancer types, including ovarian cancer [5], hepatic carcinoma [6, 7], hepatoblastoma [8], renal cell carcinoma [9], gallbladder carcinoma [10], gastric cancer [11], breast cancer [12], and multiple myeloma [13]. The contribution of CRNDE to glioma pathogenesis and progression has been recently examined. Strikingly, among 129 lncRNAs differentially expressed in gliomas, CRNDE was shown to be the most unregulated one [14]. Preliminary evidence indicated that CRNDE could interact with chromatin-modifying complexes in the epigenetic regulation of gene expression, and may enhance glioma development and malignancy via the mTOR, insulin/IGF, or EGFR signaling pathways, as well as by regulating the expression of miR-186 or miR-384 [15–19]. Although these data suggest a significant role for CRNDE in glioma growth and progression, in-depth analyses of the underlying mechanisms have yet to be undertaken.

Increasing evidence has shown that lncRNAs may act as competing endogenous RNAs(ceRNAs) that sequester miRNAs and hinder them from binding to their mRNA targets, thus altering the expression of the corresponding proteins [20]. The present study sought to gain insights into the functional roles of CRNDE in glioma. A bioinformatics database (starBase v2.0) was examined to predict miRNA binding sites in the CRNDE sequence, and miR-136-5p was identified as a putative CRNDE-binding miRNA [21]. Further bioinformatics analyses using TargetScan, miRanda, and DAVID identified Bcl-2 and Wnt2 as downstream targets of miR-136-5p in gliomas [22, 23]. Then, we conducted a series of systematic *in vitro* experiments in human glioma cells to infer molecular interactions from expression data derived from overexpression and silencing of CRNDE and miR-136-5p. Our results suggest that CRNDE functions as a ceRNA to promote glioma malignancy by indirectly inducing Bcl-2 and Wnt2 expression through binding and repression of miR-136-5p.

## RESULTS

### CRNDE is upregulated in glioma specimens and cells

**Figure 1:**
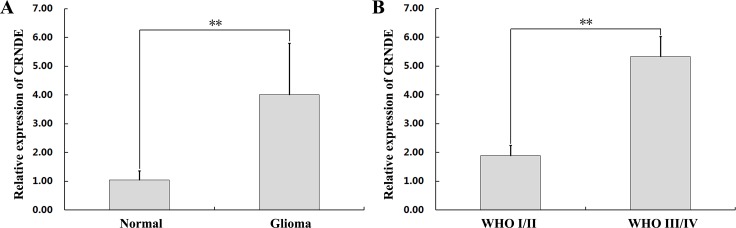
CRNDE upregulation in human glioma specimens **(A)** CRNDE levels in 47 clinical glioma specimens and 9 normal brain samples, assessed by qRT-PCR. ^**^P< 0.01. **(B)** CRNDE levels in WHO I/II and WHO III/IV glioma specimens assessed by qRT-PCR. Data are presented as mean ± SD. ^**^P< 0.01.

**Figure 2:**
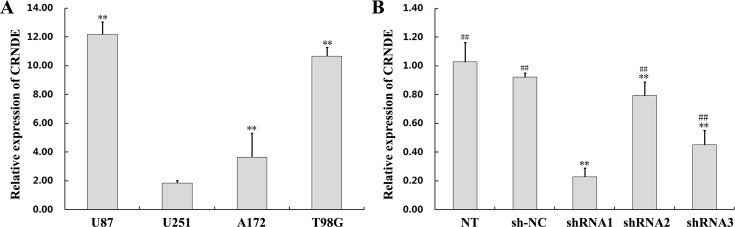
CRNDE overexpression and silencing in glioma cells **(A)** Assessment by qRT-PCR of CRNDE expression in four cell lines (U87, U251, A172, and T98G) compared with normal brain tissue. Data are presented as mean ± SD (n = 3, each group). ^**^P < 0.01 vs. U251 group. **(B)** Decreased CRNDE levels in U87 cells transfected with CRNDE shRNAs. Data are presented as mean ± SD (n = 3, each group). NT, non-transfected cells. NC, negative control. sh-NC, shRNA negative control. ^**^P< 0.01 vs. sh-NC group. ^##^*P* < 0.01 vs. shRNA1 group.

To investigate the relevance of CRNDE in glioma development, we first used qRT-PCR to determine CRNDE expression levels on specimens from 47 glioma patients. Results showed that CRNDE transcripts were dramatically upregulated in tumor samples, compared with normal brain tissues (Figure 1A). Next, CRNDE expression was further measured in high-grade and low-grade glioma specimens and in four human glioma cell lines (U87, U251, A172, and T98G). CRNDE expression was significantly higher in patients with high-grade (WHO grades III/IV), compared with both low-grade (WHO grades I/II) glioma and control samples (Figure 1B). In addition, compared to normal brain specimens, CRNDE was also upregulated in all four glioma cell lines. Among these, the U87 cell line expressed relatively high CRNDE levels, whereas relatively low CRNDE expression was detected in U251 cells (Figure 2A). Therefore, the U87 and U251 cell lines were selected for further studies assessing the functional role of CRNDE.

### CRNDE knockdown inhibits proliferation, migration, and invasion, and promotes apoptosis in glioma cells

**Figure 3:**
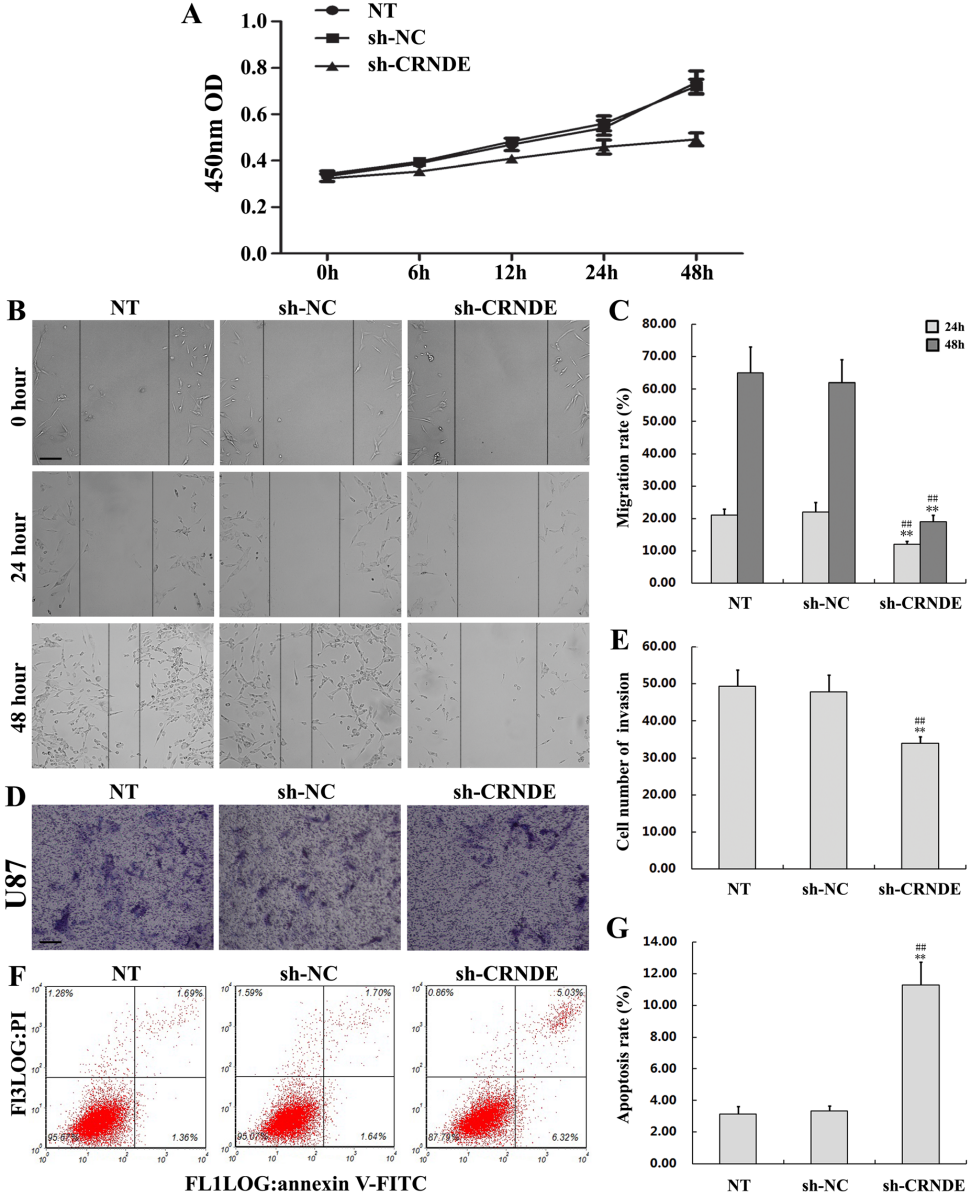
CRNDE knockdown inhibits proliferation, migration, and invasion, and promotes apoptosis in glioma cells **(A)** Effects of sh-CRNDE and sh-NC transfection on U87 cell proliferation. **(B, C)** The scratch-wound healing assay was used to assess the migration potency of U87 cells after transfection with sh-CRNDE or sh-NC. Wound closure was measured at 24 and 48 h. Representative images and accompanying statistical plots are presented. Data are presented as mean ± SD (n = 3, each group). Scale bars represent 100μm. ^**^P< 0.01 vs. NT group. ^##^*P*< 0.01 vs. sh-NC group. **(D, E)** Matrigel invasion assay results in U87 cells transfected with sh-CRNDE or sh-NC. Representative images and accompanying statistical plots are presented. Data are presented as mean ± SD (n = 5, each group). Scale bars represent 50μm. ^**^P< 0.01 vs. NT group. ^##^*P*< 0.01 vs. sh-NC group. **(F, G)** Flow cytometry determination of apoptosis in U87 cells transfected with sh-CRNDE or sh-NC. Representative images and accompanying statistical plots are presented. Data are presented as mean ± SD (n = 3, each group). ^**^P< 0.01 vs. NT group. ^##^*P*< 0.01 vs. sh-NC group. NT, non-transfected cells. NC, negative control. sh-NC, shRNA negative control; sh-CRNDE, shRNA1 targeting CRNDE.

To investigate the effect of CRNDE on proliferation, migration, invasion, and apoptosis of glioma cells, U87 cells were transfected with a shRNA (shRNA1) targeting CRNDE (sh-CRNDE) to knockdown this lncRNA. Non-transfected and sh-NC-transfected U87 cells served as controls. Gene silencing efficacy was analyzed using qRT-PCR (Figure 2B). The CCK8 assay showed that cell proliferation was markedly lower in the sh-CRNDE group than in the control group (Figure 3A). The wound-healing assay revealed that the migration rate in sh-CRNDE-transfected cells declined relative to those of the control group (Figure 3B and 3C). In addition, sh-CRNDE transfection significantly attenuated cell invasion, assessed by the Matrigel invasion assay (Figure 3D and 3E).

Flow cytometry analysis was next applied to quantify apoptosis. Results showed increased apoptosis in the sh-CRNDE group (11.27%±1.48%) compared with the sh-NC group (3.32% ± 0.34%; *P*< 0.01) (Figure 3F and 3G).

### CRNDE overexpression promotes proliferation, migration, and invasion, and inhibits apoptosis in glioma cells

**Figure 4:**
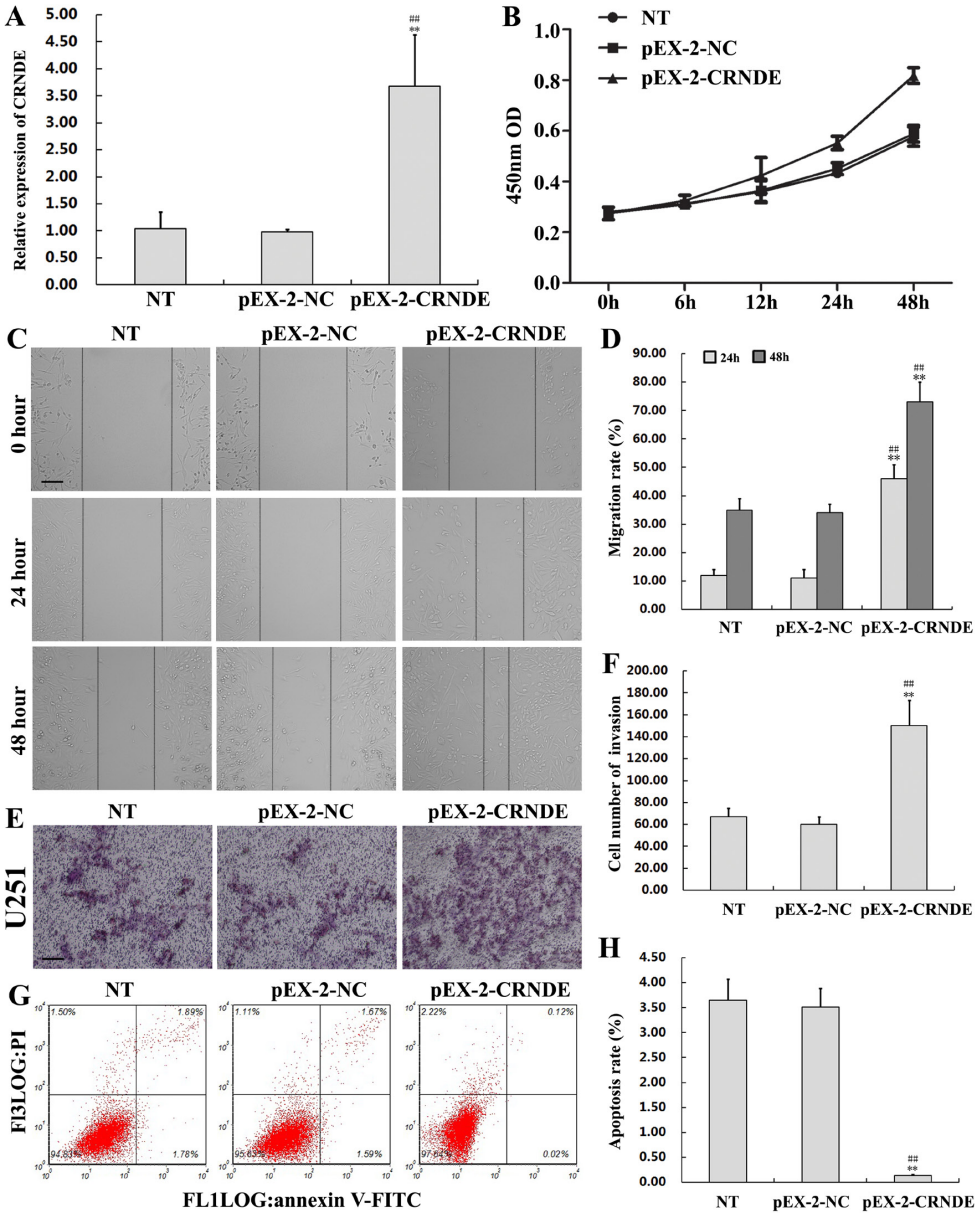
CRNDE overexpression promotes proliferation, migration, and invasion, and inhibits apoptosis in glioma cells **(A)** Increased CRNDE levels in U251 cells after transfection with pEX-2-CRNDE. Data are presented as mean ± SD (n = 3, each group). ^**^P< 0.01 vs. NT group. ^##^ *P* < 0.01 vs. pEX-2-NC group. **(B)** Effects of transfection with pEX-2-CRNDE or pEX-2-NC on the proliferation of U251 cells. **(C, D)** The wound healing assay was used to assess migration capacity in U251 cells transfected with pEX-2-CRNDE or pEX-2-NC. Wound closure was measured at 24 and 48 h. Representative images and accompanying statistical plots are presented. Data are presented as mean ± SD (n = 3, each group). ^**^P< 0.01 vs. NT group. ^##^ *P <* 0.01 vs. pEX-2-NC group. Scale bars represent 100μm. **(E, F)** Matrigel invasion assay in U251 cells transfected with pEX-2-CRNDE or pEX-2-NC. Representative images and accompanying statistical plots are presented. Data are presented as mean ± SD (n = 5, each group). ^**^P< 0.01 vs. NT group. ^##^ *P*< 0.01 vs. pEX-2-NC group. Scale bars represent 50μm. **(G, H)** Apoptosis assay by flow cytometry inU251 cells transfected with pEX-2-CRNDE or pEX-2-NC. Representative images and accompanying statistical plots are presented. Data are presented as mean ± SD (n = 3, each group). ^**^P< 0.01 vs. NT group. ^##^ *P <* 0.01 vs. pEX-2-NC group. NT, non-transfected cells. NC, negative control. pEX-2-NC, pEX2 negative control plasmid; pEX-2-CRNDE, CRNDE full length plasmid.

To further ascertain the role of CRNDE on glioma malignancy, U251 cells were transfected with pEX-2-CRNDE to upregulate CRNDE expression (Figure 4A). Non-transfected and pEX-2-NC-transfected U251 cells served as controls. Compared with the control groups, enhanced cell proliferation (Figure 4B), migration (Figure 4C and 4D), and invasion (Figure 4E and 4F) were seen upon overexpression of CRNDE. On the other hand, flow cytometry results showed a decreased rate of apoptosis in pEX-2-CRNDE-transfected cells (0.14% ± 0.02%) compared with the control pEX-2-NC group (3.65% ± 0.42%; *P*<0.01) (Figure 4G and 4H). These results are consistent with an oncogenic role of CRNDE as facilitator of proliferation, migration, invasion, and survival of glioma cells.

### CRNDE binds to miR-136-5p and negatively regulates its expression

**Figure 5:**
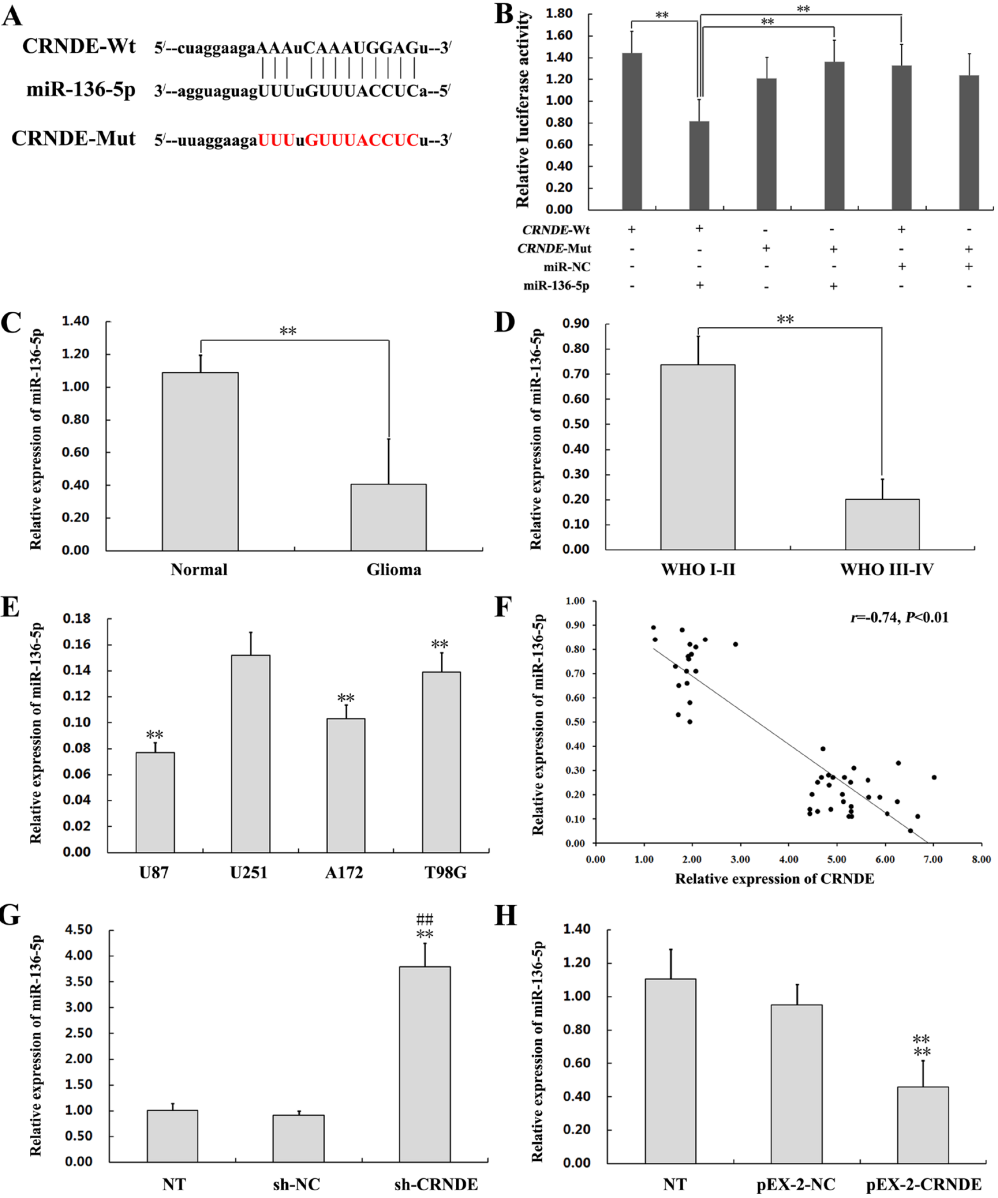
CRNDE binds to miR-136-5p and negatively regulates its expression **(A)** Schematic diagram of the predicted binding sites between CRNDE and miR-136-5p, and mutation of the putative miR-136-5p binding sequence in CRNDE. The predicted miR-136-5p binding sites in the 3′-UTR of CRNDE (CRNDE-Wt) and the designed mutant sequence (CRNDE-Mut) are indicated. The mutant sequences are labeled in red. Wt, wild type. Mut, mutant type. **(B)** A dual luciferase reporter plasmid containing CRNDE-Wt or CRNDE-Mut was co-transfected into HEK293T cells along with miR-136-5p mimics or miR-NC, and firefly vs Renilla luciferase activities were measured to compare gene expression. ANOVA was used to analyze three independent experiments. Data are presented as mean ± SD (n=3, each group). ^**^P< 0.01. **(C)** Determination of miR-136-5p expression by qRT-PCR in 47 clinical glioma specimens and 9 normal brain tissues. Data are presented as mean ± SD. ^**^P< 0.01. **(D)** Expression of miR-136-5p in WHO I/II and WHO III/IV glioma specimens, assessed by qRT-PCR. Data are presented as mean ± SD. ^**^P< 0.01. **(E)** Expression of miR-136-5p, assessed by qRT-PCR, in four glioma cell lines (U87, U251, A172 and T98G) and in normal brain tissue. ^**^P < 0.01 vs. U251 group. **(F)** Inverse correlation between CRNDE and miR-136-5p expression in 47 clinical glioma specimens (Spearman's correlation analysis; *r*=−0.74, *P*<0.01). **(G)** Expression of miR-136-5p as determined by qRT-PCR in U87 cells transfected with sh-CRNDE or sh-NC. Data are presented as mean ± SD (n = 3, each group). ^**^P< 0.01 vs. NT group. ^##^*P*< 0.01 vs. sh-NC group. **(H)** Expression of miR-136-5p as determined by qRT-PCR in U87 cells transfected with pEX-2-CRNDE or pEX-2-NC. Data are presented as mean ± SD (n = 3, each group). ^**^P< 0.01 vs. NT group. ^##^ *P <* 0.01 vs. pEX-2-NC group.

To test the hypothesis that CRNDE acts as a ceRNA, we first accessed the bioinformatics database starBase v2.0 to search for potential CRNDE/miRNA interactions. The results predicted that miR-136-5p can bind the lncRNA product of the CRNDE gene (Figure 5A and Table 1). To verify this prediction, we generated wild type (Wt) CRNDE luciferase plasmids containing potential miR-136-5p binding sites, as well as mutant variants of each site. These plasmids were co-transfected with miR-136-5p mimics or miR-NC into HEK293T cells, and then luciferase assays were performed. As shown in Figure 5B, luciferase activity in the pEX-2-CRNDE Wt+miR-136-5p group was lower than in the pEX-2-CRNDE Wt group, the pEX-2-CRNDE-Mut+miR-136-5p group, and the pEX-2-CRNDE-Wt+miR-NC group(*P*<0.01). These results indicated that miR-136-5p could suppress the activity of a luciferase reporter harboring CRNDE-Wt, and does not affect CRNDE-Mut activity. Furthermore, compared to normal brain tissues, the expression of miR-136-5p was significantly downregulated in the 47 clinical glioma specimens and in our four glioma cell lines, and was also significantly and negatively correlated with pathological grade of glioma (Figures 5C-5E). Moreover, Spearman's correlation analysis showed that CRNDE and miR-136-5p levels were inversely correlated in glioma samples(*r* =−0.74, *P*<0.01) (Figure 5F). This relationship was examined experimentally in glioma cells transfected with either pEX-2-CRNDE or sh-CRNDE. Results of qRT-PCR showed that miR-136-5p expression was upregulated in U87 cells transfected with sh-CRNDE and downregulated in U251 cells transfected with pEX-2-CRNDE (Figure 5G and 5H). Taken together, these data suggest that CRNDE binds to miR-136-5p and negatively regulates its expression in glioma cells.

**Table 1:** StarBase (v2.0) predicted the miRNA that target CRNDE

MiRNA Name	mir Accession	Target Name	Target Location	Bio Complex	Clip Read Num	Gene Name	Target Sites
miR-136-5p	MIMAT0000448	CRNDE	chr16:54957644-54957666 [-]	1	8	CRNDE	1

### Overexpression of miR-136-5p inhibits proliferation, migration, and invasion, and promotes apoptosis in glioma cells

**Figure 6:**
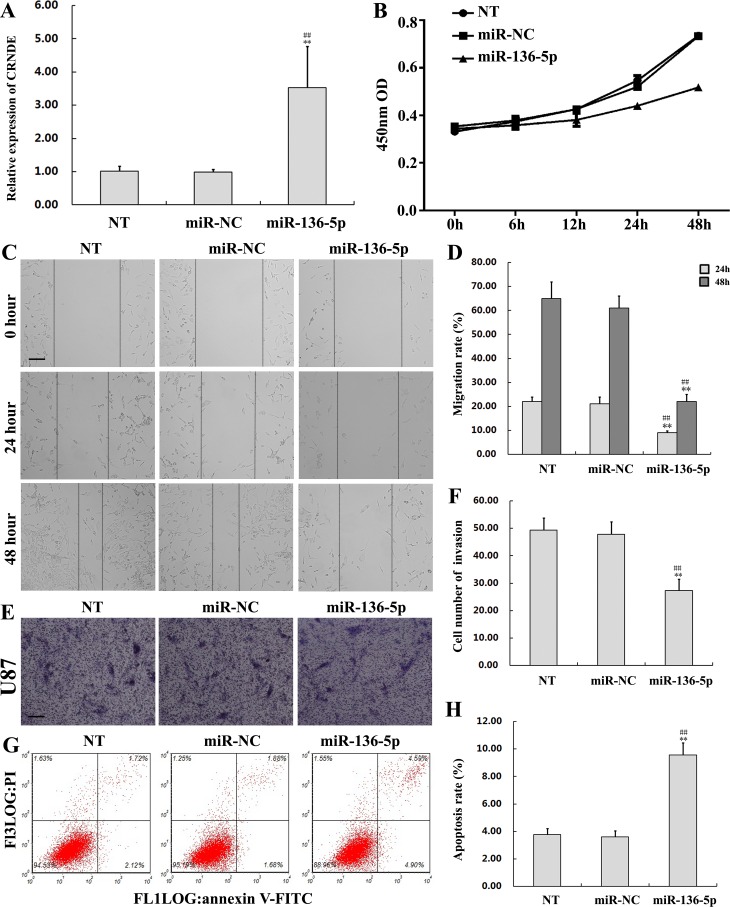
Overexpression of miR-136-5p inhibits proliferation, migration, and invasion, and promotes apoptosis in glioma cells **(A)** miR-136-5p levels in U87 cells transfected with miR-136-5p mimics. Data are presented as mean ± SD (n = 3, each group).^**^P< 0.01 vs. NT group.^##^*P*< 0.01 vs. miR-NC group. **(B)** Proliferation assay results in U87 cells transfected with miR-136-5p mimics or miR-NC. **(C, D)** Migratory capacity of U87 cells transfected with miR-136-5p mimics or miR-NC. Wound closure was measured at 24 and 48 h. Representative images and accompanying statistical plots are presented. Data are presented as mean ± SD (n = 3, each group). ^**^P< 0.01 vs. NT group.^##^*P*< 0.01 vs. miR-NC group. Scale bars represent 100μm. **(E, F)** Results of the Matrigel invasion assay in U87 cells transfected with miR-136-5p mimics or miR-NC. Representative images and accompanying statistical plots are presented. Data are presented as mean ± SD (n = 5, each group). ^**^P< 0.01 vs. NT group.^##^*P*< 0.01 vs. miR-NC group. Scale bars represent 50μm. **(G, H)** Apoptosis assay by flow cytometry inU87 cells transfected with miR-136-5p mimics or miR-NC. Representative images and accompanying statistical plots are presented. Data are presented as mean ± SD (n = 3, each group). ^**^P< 0.01 vs. NT group.^##^*P*< 0.01 vs. miR-NC group. NT, non-transfected cells. NC, negative control. miR-NC, negative control miR plasmid; miR-136-5p, miR-136-5p mimics.

To further explore the function of miR-136-5p in glioma, U87 cells were transfected with miR-136-5p mimics to upregulate miR-136-5p expression (Figure 6A). Non-transfected, and miR-NC-transfected cells served as controls. The CCK8 assay showed reduced proliferation in U87 cells transfected with miR-136-5p mimics, compared with control cells (Figure 6B). On the other hand, the migration and invasion assays revealed that miR-136-5p overexpression weakened both the migratory and invasive potential of glioma cells (Figures 6C-6F). In addition, transfection with miR-136-5p mimics increased apoptosis (9.57% ± 0.88% vs 3.61% ± 0.44% in the miR-NC group; *P*< 0.01) (Figure 6G and 6H). This evidence indicates that miR-136-5p exerts a tumor suppressor role in glioma.

### CRNDE promotes glioma malignancy by preventing miR-136-5p-mediated downregulation of Bcl-2 and Wnt2

**Figure 7:**
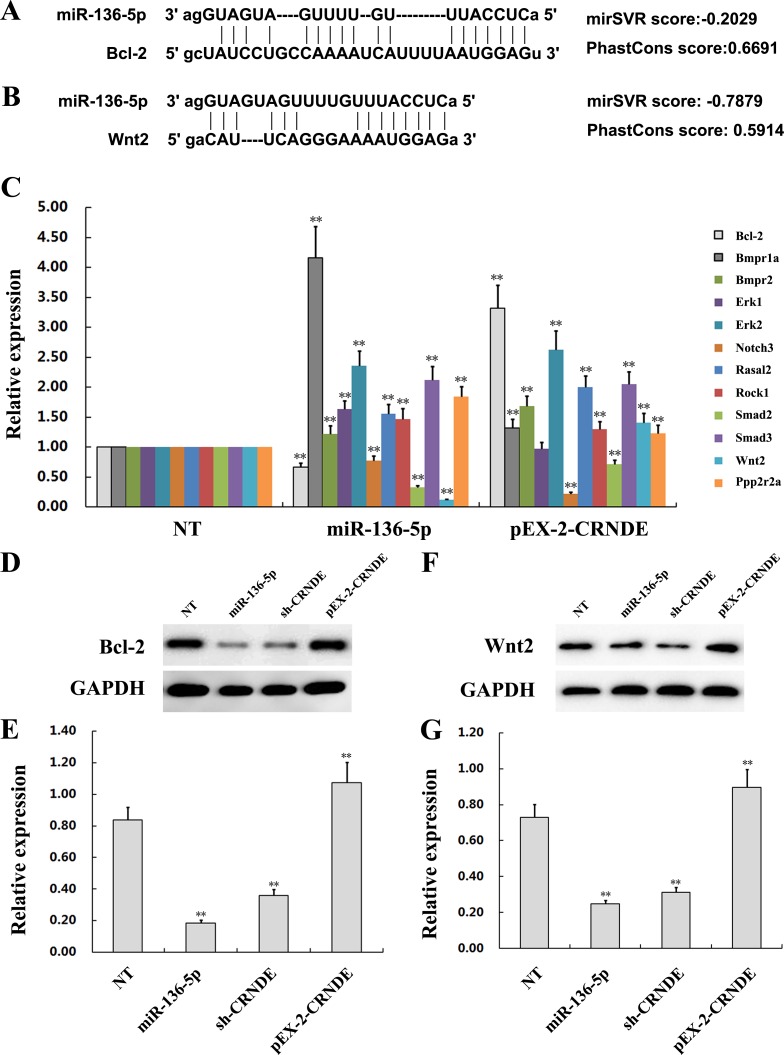
The lncRNA CRNDE increases Bcl-2 and Wnt2 expression by inhibiting miR-136-5p **(A)** Schematic diagram of the predicted binding sites between miR-136-5p and Bcl-2. **(B)** Schematic diagram of the predicted binding sites between miR-136-5p and Wnt2. **(C)** Expression of downstream target genes of miR-136-5p in U251 cells transfected with pEX-2-CRNDE and in U87 cells transfected with miR-136-5p mimics, compared with non-transfected (NT) cells. Representative images and accompanying statistical plots are presented. Data are presented as mean ± SD (n = 3, each group). ^**^P< 0.01 vs. NT group. **(D-G)** Expression of Bcl-2 and Wnt2, as determined by western blot, in U251 cells transfected with pEX-2-CRNDE and in U87 cells transfected with sh-CRNDE or miR-136-5p mimics. NT cells were tested for comparison. Representative images and accompanying statistical plots are presented. Data are presented as mean ± SD (n = 3, each group). ^**^P< 0.01 vs. NT group.

**Figure 8:**
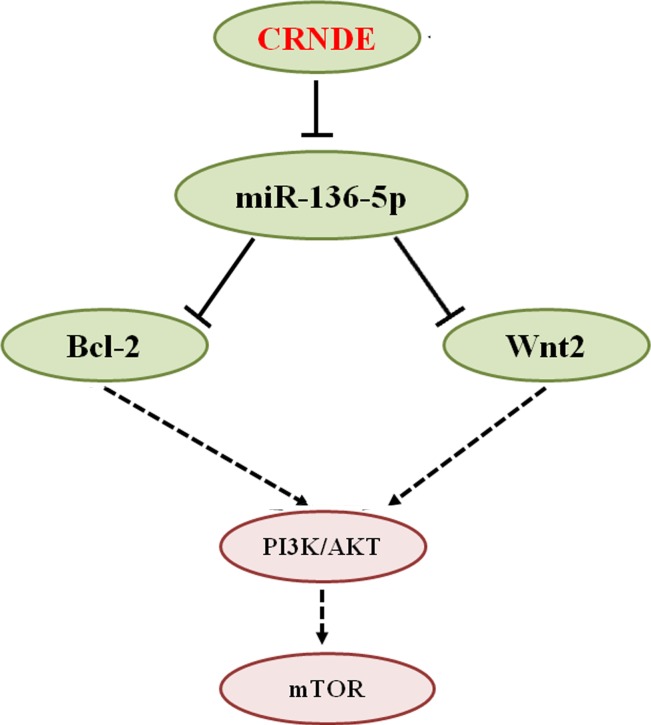
Schematic representation of CRNDE actions in glioma Based on our present study and previous reports, we conclude that CRNDE promotes glioma progression by competitively binding miR-136-5p and inhibiting the post-transcriptional repression of Bcl-2 and Wnt2 mediated by this miRNA. This leads to upregulation of Bcl-2 and Wnt2 and activation of the PI3K/AKT/mTOR signaling pathway.

We performed a bioinformatics analysis on TargetScan, miRanda, and DAVID and identified several genes, among them Bcl-2 and Wnt2, as candidate targets of miR-136-5p (Figure 7A and 7B). To determine whether CRNDE acted as a ceRNA by sequestering miR-136-5p and hindering it from binding to its target mRNAs, the expression of candidate miR-136-5p targets was examined by qRT-PCR in U251 cells transfected with pEX-2-CRNDE and in U87 cells transfected with miR-136-5p mimics or sh-CRNDE. Results showed that among the several genes targeted by miR-136-5p, the expression of Bcl-2 and Wnt2 was significantly decreased in U87 cells transfected with miR-136-5p mimics. Conversely, the expression of both genes was increased in U251 cells transfected with pEX-2-CRNDE (Figure 7C). To evaluate whether these changes were accompanied by corresponding variations in protein expression, Bcl-2 and Wnt2 levels were determined by western blot. As shown in Figures 7D-7G, protein expression results paralleled the findings obtained by qRT-PCR, namely, upregulation of Bcl-2 and Wnt2 in pEX-2-CRNDE-transfected cells and downregulation of these proteins in U87 cells transfected with either sh-CRNDE or miR-136-5p mimics. These results suggest that CRNDE promotes glioma aggressiveness via suppression of miR-136-5p-mediated downregulation of Bcl-2 and Wnt2 (Figure 8).

## DISCUSSION

Glioma, a devastating invasive cerebral tumor, is the leading cause of central nervous system tumor-related death in adults, and its early diagnosis and treatment remain difficult medical problems. Research has shown that some lncRNAs, which represent a prominent subtype of regulatory ncRNA, are closely associated with glioma pathogenesis and progression [24–26], and may be useful biomarkers and therapeutic targets for glioma diagnosis and treatment. The present study suggests an essential role for the CRNDE-miR-136-5p-Bcl-2/Wnt2 regulatory pathway in the malignant progression of glioma, by showing that CRNDE competitively binds miR-136-5p and thus indirectly inhibits the post-transcriptional expression of Bcl-2 and Wnt2, whose upregulation is known to be associated with glioma malignancy. The ceRNA/miRNA/mRNA regulatory pathway concept not only provides a novel avenue to study the molecular mechanisms of glioma biology, but also encourages the establishment of specific and sensitive expression profiles of lncRNA/miRNA interactions that may aid in early diagnosis and targeted treatment of glioma.

Some clues regarding the roles of CRNDE on glioma pathogenesis and progression have recently been revealed. Zhang *et al.* analyzed microarray data of glioma samples retrieved from the GEO database and identified 129 differentially expressed lncRNAs. Notably, among these, CRNDE showed a 14-32-fold increase compared to normal brain tissues [14]. The overexpression pattern of CRNDE in glioma was also confirmed by high throughput microarray data and revealed a potential role in the malignant properties of glioma [24]. Moreover, a positive correlation between CRNDE expression and glioma grade and recurrence has been reported [27].

Interestingly, during the completion of the present manuscript Zheng *et al.* reported that elevated CRNDE expression promotes cell proliferation, invasion, and migration, and inhibits apoptosis of glioma cells through negative regulation of miR-384 and miR-186 [18, 19]. This evidence, as well as the present results highlight the relevance of CRNDE in glioma, by implying that a single lncRNA controls the activity of multiple miRNAs, conceivably affecting the expression of a large number of genes.

Other mechanisms have been proposed to also underlie the oncogenic role of CRNDE in glioma. Khalil *et al.* reported that CRNDE interacts with chromatin-modifying complexes to affect the epigenetic regulation of gene expression or to facilitate its regulation by insulin/IGF signaling [28]. Subsequently, preliminary evidence suggested that CRNDE enhances glioma malignancy via stimulation of the mTOR signaling pathway [15]. Recently, Kiang *et al.* reported that aberrant expression of CRNDE mediated by EGFR signaling might contribute significantly to glioma genesis [17]. Thus, these studies suggest that CRNDE acts as an oncogene in glioma cells via different mechanisms.

Our study confirmed that CRNDE expression is upregulated in clinical samples, and established its correlation with disease grade. Then, we detected that CRNDE expression is unregulated in four human glioma cell lines (U87, U251, A172, and T98G); compared to normal brain tissues, U87 cells had the highest CRNDE expression, whereas U251cells had the lowest. Therefore, U251 cells were chosen for CRNDE overexpression experiments, while U87cells were chosen to assess the effects of CRNDE silencing. This design allowed us to assess more clearly the potential contribution of CRNDE to glioma cell proliferation, migration, invasion, and survival, while also ensuring that the experimental results would not be limited to a single cell line. Upon transfection of U87 cells with sh-CRNDE significant attenuation of proliferation, invasion, and migration, as well as increased apoptosis paralleled the reduction in CRNDE expression. Meanwhile, CRNDE overexpression had opposite effects. These data are consistent with previous studies indicating an oncogene-like role for CRNDE in the pathogenesis and development of glioma [15, 19].

Because off-target effects are a common phenomenon in shRNA experiments, we completed a further series of confirmatory experiments with a second shRNA (shRNA3) targeted to CRNDE and a scrambled shRNA control. The results, summarized in Supplementary Figure 1, allow us to conclude that our knockdown experiments are specific and convincing, as different shRNAs targeting CRNDE induced similar changes in gene expression profiles and phenotypes.

Our results indicate that CRNDE supports glioma progression by acting as a ceRNA. The ceRNA hypothesis, proposed by Salmena *et al.*, states that lncRNAs (and other RNAs) compete with miRNA for binding to the target mRNA, thus preventing its degradation by miRNA and indirectly promoting target gene expression [20]. The ceRNA hypothesis provided a network regulatory model for ceRNA-miRNA-mRNA interactions, underlining a new point of view for the elucidation of tumor pathogenesis. For instance, Gao *et al.* discovered that a lncRNA, RoR, was capable of binding to miR-145 for competitive regulation of Nanog expression in pancreatic cancer [29], while the lncRNA HOTAIR was shown to act as a ceRNA for miR-217 to facilitate HIF-1α expression and upregulation of AXL in renal cell carcinoma [30]. Indeed, substantial evidence provided by diverse research groups indicates that the ceRNA phenomenon has universality and plays an extensive role in biological processes [31, 32].

We searched for potential binding sites of CRNDE to miRNAs on bioinformatics databases and found a putative, specific binding site for miR-136-5p. Such interaction was confirmed by dual luciferase activity assays. Meanwhile, experiments in human glioma cells showed that CRNDE not only binds miR-136-5p but also reduces its expression, which is consistent with the inverse correlation between CRNDE and miR-136-5p expression observed in clinical glioma specimens. *in vitro* data showed that miR-136-5p overexpression inhibited tumor cell proliferation, migration, and invasion, and enhanced apoptosis, implying that miR-136-5p exerts a tumor suppressive function in glioma cells. The involvement of miR-136-5p in tumor development has been studied by many groups, and diverse mechanisms have been proposed. Reports have shown that downregulation of miR-136-5p in human gliomas is significantly associated with a more aggressive and poor prognostic phenotype; conversely, its overexpression promotes apoptosis and modulates sensitivity to cisplatin by targeting AEG-1, Bcl-2, and E2F1 [33–35]. Consequently, we run bioinformatics prediction analyses on TargetScan, miRanda, and DAVID, which identified Bcl-2 and Wnt2 as downstream target genes of miR-136-5p. These genes attracted our attention because Bcl-2 has been reported to be a direct target of miR-136-5p [33], while both Wnt2 and Bcl-2 have been linked to the pathogenesis of glioma [36, 37]. Wnt2 is a key activator of Wnt pathway, an important regulator of cell proliferation, development, and differentiation [38]. Increasing evidence suggests that aberrant activation of Wnt signaling is involved in glioma development and progression [39]. Pu *et al.* reported that downregulation of Wnt2 suppresses malignant glioma cell growth associated with decreased activation of the of PI3K/AKT signaling pathway [36], which affects cell growth and survival through modulation of mammalian target of Rapamycin (mTOR) signaling [40]. Furthermore, because apoptosis is a critical cellular process in regulating cancer cell growth, we focused on the detection of apoptosis-related gene dysregulation. Bcl-2 is a classical apoptosis suppressor gene, stabilizing the mitochondrial membrane, and inhibiting the release of cytochrome C and apoptosis suppressor factors. Bcl-2 is also a downstream target of the PI3K/AKT signaling pathway [41, 42].

Our *in vitro* experiments in glioma cells confirmed that changes in the expression of CRNDE and miR-136-5p result in changes in Wnt2 and Bcl-2 expression at the mRNA and protein levels, although our study did not provide direct evidence for the corresponding miRNA/mRNA interactions.

In summary, although glioma cell growth and invasion has been previously linked to CRNDE-mediated modulation of mTOR signaling [15, 26], we expanded this knowledge through experiments that indicated that CRNDE could increase, via repression of miR-136-5p, the levels of Bcl-2 and Wnt2, two downstream effectors of the PI3K/AKT/mTOR signaling pathway, thus contributing to the malignant characteristics of glioma (Figure 8). Future studies using RNA pull-down and mass spectrometry should evaluate the CRNDE interactome to fully elucidate its contributory role to the progression of glioma and other tumors.

## MATERIALS AND METHODS

### Human glioma specimens

Human glioma specimens were obtained from patients undergoing initial surgery who were diagnosed with glioma at the Department of Neurosurgery, Anhui Provincial Hospital, from February 2014 to November2016. None of the patients had received chemotherapy or radiotherapy prior to surgery. After surgical resection, parts of the fresh glioma specimens were sent for routine neuropathological evaluation, and the remaining tumor was immediately frozen and preserved in liquid nitrogen. The research methods and procedures were approved by the Ethics Committee of Anhui Provincial Hospital, and written informed consent was obtained from all patients. Glioma grades were determined by three neuropathologists according to the 2007 World Health Organization (WHO) classification. Eighteen cases were classified as grade I/II (low-grade) and 29 cases as grade III/IV (high-grade). Nine samples of normal brain tissue obtained from patients with severe traumatic brain injury who underwent decompression surgery were used as negative control.

### Cell culture and transfection

Human U87 and U251 glioma cell lines were purchased from the Beijing North Carolina Souren Biotechnology Research Institute (Beijing, China). The human A172 and T98Gglioma cell lines were kind gifts from Dr. Suling Liu (University of Science and Technology of China, Anhui, China). Human embryonic kidney (HEK) 293T cells were purchased from the Chinese Academy of Sciences Type Culture Collection(Shanghai, China). Cells were cultured in RPMI 1640 (HyClone, UT, USA) supplemented with 10% fetal bovine serum (FBS, Gibco, NY, USA). All cells were cultured in a humidified incubator at 37 °C with 5% CO_2_.

Plasmids encoding specific short-hairpin RNAs against CRNDE(sh-CRNDE), full-length CRNDE (pEX-2-CRNDE), or miR-136-5p (miRNA mimics), or their respective scrambled, non-targeting sequences, i.e. short-hairpin negative control (sh-NC), pEX-2 negative control (pEX-2-NC), and miR negative control (miR-NC) were designed and synthesized by GenePharma (Shanghai, China). These sequences are listed in Table 2. Cells were seeded into 12-well plates until they were at 80% confluence, and transfection was carried out using Lipofectamine 2000 (Invitrogen) according to the manufacturer's instructions. After 6h of transfection, the medium was replaced with RPMI 1640 medium containing 10%FBS. The efficiency of knockdown and upregulation was analyzed using qRT-PCR.

**Table 2:** Sequences of primers and shRNAs

Name	Sequence (5′-3′)
CRNDE	Forward: 5′-ACACGGCTTTCCGGAGTAGA-3′
	Reverse: 5′-GCCAACATTTGGAGGAACCC-3′
mir-136-5p	Forward: 5′-ACTCCATTTGTTTTGATGATGGA-3′
	Reverse: Common Reverse primer in kit
Smad2	Forward: 5′-TTGATGGTCGTCTCCAGGTAT-3′
	Reverse: 5′-AAGTTCTGTTAGGATCTCGGTGT-3′
Smad3	Forward: 5′-GAACGGGCAGGAGGAGAAAT-3′
	Reverse: 5′-CAGGGACCTGGGGATGGT-3′
Ppp2r2a	Forward: 5′-GAGCTGGAGGAGGGAATGATAT-3′
	Reverse: 5′-CTCCTGCTCCTGTTGAAAGATG-3′
Notch3	Forward: 5′-GTATCTGCACCAACCTGGCA-3′
	Reverse: 5′-GTTCAGGCATGGGTTGGGG-3′
Rasal2	Forward: 5′-AAAAGAGTCACGTTCCCATGAAT-3′
	Reverse: 5′-CCAAGGATGCTACTATGAAGTGG-3′
Bcl-2	Forward: 5′-CTGGGATGCCTTTGTGGAAC-3′
	Reverse: 5′-GCAGGCATGTTGACTTCACT-3′
Rock1	Forward: 5′-CTGGTGACCCTGAGGTTCCG-3′
	Reverse: 5′-GCATCCAATCCATCCAGCAAA-3′
Bmpr1a	Forward: 5′-TCGTTCAAGGACAGAATCTGGA-3′
	Reverse: 5′-GGCAAGGTATCCTCTGGTGCTA-3′
Bmpr2	Forward: 5′-TGGCAGCAGTATACAGAGTGA-3′
	Reverse: 5′-ACTGCCCTGTTACTGCCATT-3′
Erk1	Forward: 5′-GCAACACCACCTGCGACCTT-3′
	Reverse: 5′-CGTAGCCACATACTCCGTCA-3′
Erk2	Forward: 5′-ATTTGTCAGGACAAGGGCTC-3′
	Reverse: 5′-TCCAAACGGCTCAAAGGAGT-3′
Wnt2	Forward: 5′-AAGAAGATGGGAAGCGCCAA-3′
	Reverse: 5′-ACCGCTTTACAGCCTTCCTG-3′
GAPDH	Forward: 5′-ATGTTGCAACCGGGAAGGAA-3′
	Reverse: 5′-AGGAAAAGCATCACCCGGAG-3′
U6	Forward: 5′-CTCGCTTCGGCAGCACA-3′
	Reverse: Common Reverse primer in kit
shRNA1	GGTGTTAAGTGTGATGCTTCC
shRNA2	GGATGCTGTCAGCTAAGTTCA
shRNA3	GGAAGGAGGAGATTCTGAAGA

### RNA isolation and quantitative real-time polymerase chain reaction (qRT-PCR)

Total RNA and miRNA were extracted from glioma specimens, normal brain tissues, and cells using the Ultrapure RNA Kit (DNase I; CWBIO, China) and the miRcute miRNA Kit(TIANGEN, China) following the manufacturers’ instructions. RNA was reverse transcribed to complementary DNA (cDNA) by using the TIANScript cDNA Kit (TIANGEN, China). The miRcute miRNA First-Strand cDNA Synthesis Kit (TIANGEN, China) was used to generate cDNA from miRNA. Primers for each transcript were synthesized by Invitrogen (Shanghai, China) and are listed in Table 2. U6 and GAPDH were used as endogenous controls for miRNA and gene expression detection, respectively. The SYBR®Green Real-time PCR Kit (Takara, Liaoning, China) and the miRcute Plus miRNA qPCR Detection Kit (TIANGEN, China) were used for qRT-PCR assays using the ABI 7500 Real-Time PCR System (Applied Biosystems, CA, USA). Relative expression was normalized to that of endogenous controls using the comparative cycle threshold method, and the fold change in gene expression was calculated using the 2^−ΔΔCt^ method.

### Cell proliferation assay

Cell proliferation assays were performed using the Cell Counting Kit-8 (CCK-8, Beyotime, Jiangsu, China) based on the manufacturer's protocol. After transfection, cells were seeded into 96-well plates at an initial density of 1,000-5,000 cells per well. After 6, 12, 24, or 48h of culture, 10μL of CCK-8 was added to each well. After further incubation at 37°C for 2h, absorbance (450 nm) was measured using a spectrophotometer.

### Matrigel invasion assay

Cell invasion was quantified using Matrigel-coated transwell chambers (Corning, NY, USA) according to the manufacturer's instructions. Cells were resuspended in 100μL of serum-free medium at a density of 1×10^4^/mL and seeded into the upper chamber with 100 μL of DMEM/10% FBS added to the lower chamber. After 24h of incubation, inserts were removed from the plates and non-migrated cells on the upper side of the chamber were wiped with a cotton swab. Afterwards, the membranes were fixed with 4% paraformaldehyde for 15 min and stained with 0.5% Giemsa (Beyotime, Jiangsu, China) for 3 min. Five random fields for each membrane were chosen to count under an inverted microscope the invading cells, and images were taken for statistical analysis.

### Cell migration assay

Cells were cultured in 12-well plates. When they reached 80-90% confluence, cell layers were scratched using a rod with a 1μmdiameter tip, gently washed, and cultured for another 24or 48 h. Wound closure images were obtained at 0,24, and 48h time-points at fixed microscopic observation points, and cell migration distances were measured and analyzed using Image Pro Plus 6.0 software.

### Apoptosis assay

Apoptosis was quantified in U87 and U251 cells using an Annexin V-FITC/PI staining kit (BestBio, China). Briefly, cells from each experimental group were seeded in 6-well plates (1×10^6^cells/well) and cultured in complete medium for 48h. The cells were then washed with ice-cold phosphate buffered saline (PBS), harvested, and resuspended in 400μL of Annexin V binding buffer at a density of 1×10^6^ cells/mL. Next, 5μL of Annexin V-FITC was added to the cell suspensions, which were further incubated at 2-8°C for 15 min in the dark. After addition of 10μL of PI and incubation in the dark at 2-8°C for another 5 min. The samples were analyzed immediately by flow cytometry (Beckman Gallios, Beckman Coulter). Data were analyzed by FCS express V3 software. This assay was performed in triplicate.

### Reporter vector construction and luciferase reporter assay

Luciferase reporter plasmids (CRNDE-Wt and CRNDE-Mut) were designed and constructed by GenePharma (Shanghai, China). The sequence of the putative binding site was replaced as indicated in Figure 5A (CRNDE-Mut) to prevent normal CRNDE function. Full-length CRNDE sequences were amplified by PCR and cloned into a pmirGLO Dual-luciferase miRNA Target Expression Vector (Promega) to construct the luciferase reporter vectors. HEK-293T cells were seeded in 12-well plates, and the cells were co-transfected with luciferase reporter plasmids and miR-136-5p mimics or miR-NC plasmids usingLipofectamine2000transfection reagent when they reached 80% confluence. 48 h after transfection, firefly and Renilla luciferase activities were measured by the Dual-Luciferase Reporter Assay System Kit (Promega) to analyze the interaction between CRNDE and miR-136-5p.

### Western blot analysis

Cells and tissues were washed with cold PBS and lysed in ice-cold RIPA buffer containing protease inhibitors (Beyotime, China), followed by incubation on ice for 30 min and centrifugation (4°C, 30 min). The supernatant was collected, and protein concentration was measured. Equal amounts of protein from each sample were separated by electrophoresis on a 10% SDS-polyacrylamide gel (Beyotime, China), electrotransferred to a PVDF membrane (Millipore, USA) and blocked. Antibodies against GAPDH (Santa Cruz, 1:1000), Bcl-2 (Bioworld, 1:500), and Wnt2 (Abcam, 1:1000) were used. Immunoblots were visualized by chemiluminescence using an ECL detection system (BeyoECLPlus, Beyotime, China). The intensity of the bands was determined using Image Pro Plus 6.0 software.

### Statistical analysis

Data analysis was performed using SPSS 18.0 statistical software (SPSS Inc., Chicago, IL, USA). All results are presented as mean±standard error(SD). Differences between two groups were analyzed using Student's t test; one-way ANOVA was used when more than two groups were compared. Correlation between CRNDE and miR-136-5p expression was analyzed by Spearman's correlation analysis. *P*< 0.05 was considered statistically significant.

## SUPPLEMENTARY FIGURE



## Abbreviations

CRNDE: Colorectal neoplasia differentially expressed
sRNA: small RNA
ncRNA: non-coding RNA
lncRNA: long non-coding RNA
ceRNA: competing endogenous RNA
miRNA: microRNA
siRNA: small interfering RNA
mTOR: mammalian target of rapamycin
qRT-PCR: quantitative real-time polymerase chain reaction
NT: non-transfected
NC: negative control
Bcl-2: B-cell lymphoma-2
Wnt2: Wnt family member 2
