# Supplementary material for: The long non-coding RNA CRNDE acts as a ceRNA and promotes glioma malignancy by preventing miR-136-5p-mediated downregulation of Bcl-2 and Wnt2
Source: Oncotarget. 2017 Oct 4;8(50):88163–78. doi: 10.18632/oncotarget.21513 (PMC5675701; doi:10.18632/oncotarget.21513)
Supplement: Supplementary file 1 [file oncotarget-08-88163-s001.pdf]

The long non-coding RNA CRNDE acts as a ceRNA and promotes glioma malignancy by preventing miR-136-5p-mediated downregulation of Bcl-2 and Wnt2

SUPPLEMENTARY MATERIALS

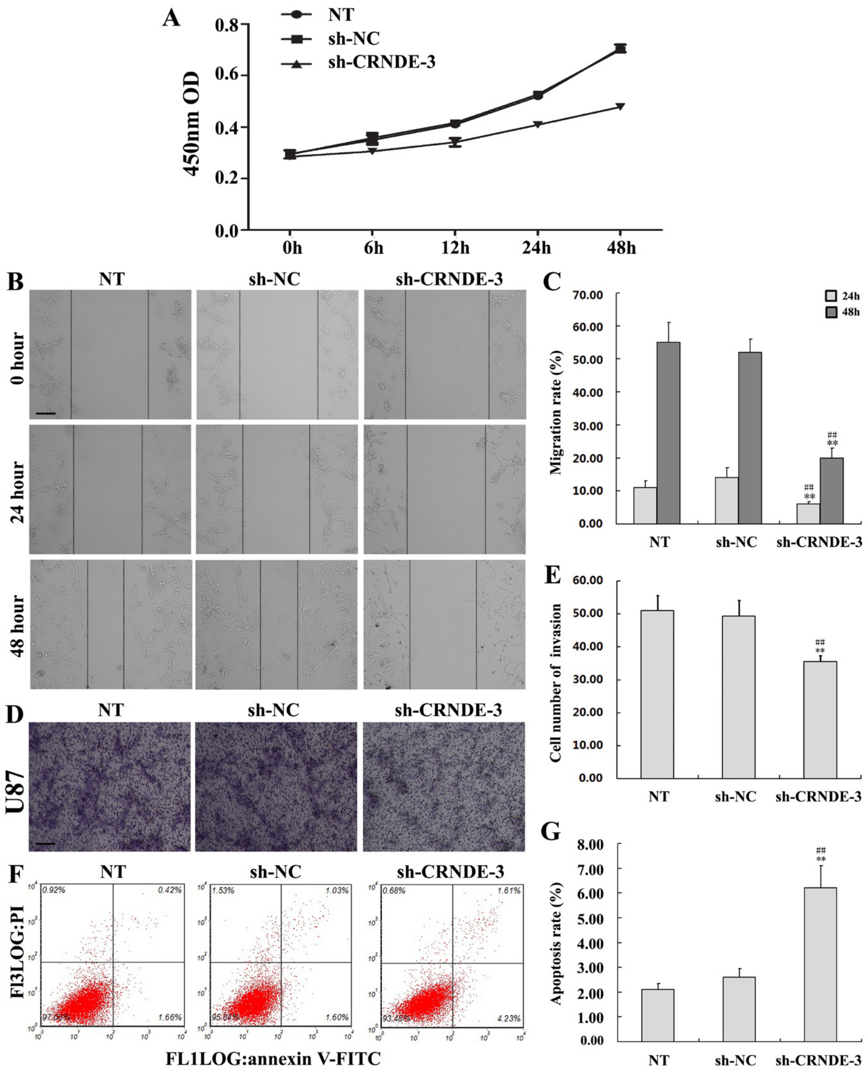

Supplementary Figure 1: CRNDE knockdown with a second shRNA(shRNA3) also inhibits proliferation, migration, and invasion, and promotes apoptosis in glioma cells. **(A)** Effects of sh-CRNDE-3 and sh-NC transfection on U87 cell proliferation.**(B, C)** The scratch-wound healing assay was used to assess the migration potency of U87 cells after transfection with sh-CRNDE-3 or sh-NC. Wound closure was measured at 24 and 48 h. Representative images and accompanying statistical plots are presented. Data are presented as mean ± SD (n = 3, each group). Scale bars represent 100μm. ***P*< 0.01 vs. NT group. $^{##}$*P*< 0.01 vs. sh-NC group. **(D, E)** Matrigel invasion assay results in U87 cells transfected with sh-CRNDE-3 or sh-NC. Representative images and accompanying statistical plots are presented. Data are presented as mean ± SD (n = 5, each group). Scale bars represent 50μm. ***P*< 0.01 vs. NT group. $^{##}$*P*< 0.01 vs. sh-NC group. **(F, G)** Flow cytometry determination of apoptosis in U87 cells transfected with sh-CRNDE-3 or sh-NC. Representative images and accompanying statistical plots are presented. Results showed increased apoptosis in the sh-CRNDE group (6.21%±0.91%) compared with the sh-NC group (2.62% ± 0.34%). Data are presented as mean ± SD (n = 3, each group). ***P*< 0.01 vs. NT group. $^{##}$*P*< 0.01 vs. sh-NC group. NT, non-transfected cells. NC, negative control. sh-NC, shRNA negative control; sh-CRNDE-3, shRNA3 targeting CRNDE.
